# On the Pathology of Toothache

**Published:** 1845-12

**Authors:** 


*On the Pathology of Toothache.*—By Dr. HEILDEN.—Toothache may depend either upon congestion, inflammation, or a lesion of innervation. 1st. *Congestion; this may have its seat either in the membrane exterior to the fang of the tooth, in that lining its central canal, or in the ganglion, which supplies the tooth with nerves.* Congestion, when seated in the lining membrane of the fang, may be known by lancinating, throbbing pains, which are increased by any excitement of the system; these pains are variable in their character, sometimes lasting but for a few minutes, and again for many hours, they are generally increased towards evening, and when the patient is in bed. The tooth, whose lining membrane is affected, is sensible to the touch, or to pressure, and frequently conveys the sensation of being somewhat above the level of the surrounding teeth. The frequent application of cold water to the affected tooth is one of best means of cure that can be adopted in this form of odontalgia. 2d. *Congestion in the lining membrane of the canal, and of the dental nerves.* Toothache dependent upon these causes may be distinguished from the variety just described, by the tooth not being painful on pressure nor conveying the sensation of prominency over its fellows. It may also be distinguished by the effect which cold water produces upon it; if the tooth be carious at the crown, cold water immediately gives relief, but if it be not so, the pain undergoes an exacerbation for some time, but under the use of the remedy it eventually disappears. Young, plethoric persons, and pregnant women, are those most subject to this form of toothache. In obstinate cases, besides the local application of cold water, it may be necessary to use the foot-bath, and administer purgatives. Where careis exists along with this form of congestion, timely plugging must be had recourse to. All stimulants, such as tinctures in common use for curing toothache, must be avoided here, as they only increase the mischief. *Inflammation.*—This process when occurring in the teeth of a healthy individual, will produce the phenomena of healthy inflammation in any other part of the body; in individuals affected with gout, rheumatism, or scrofula, it will present the specific character of these diseases. Inflammation of the internal lining membrane of the tooth-fang (periodontitis,) occurs much oftener in carious than in healthy teeth; it is characterized by a dull aching, rather than actual pain, from which the patient fancies he obtains relief by pressing his teeth strongly together. This dull aching after some time is exchanged into an acute, *boring* pain, which extends to the neighbouring teeth; at this stage, the affected tooth seems more elevated than its fellows; and this sensation prevents perfect closure of the mouth, and to a great degree interferes with mastication. In some cases this local inflammation causes severe constitutional disturbance, heat and redness of the cheeks, severe headache, and general febrile irritation. In this state, if nothing be done to check the local inflammation, this acquires greater intensity. The acute boring pain is now changed into a dull aching attended with throbbing, if the gum about the affected tooth be examined, it will be found intensely inflamed, the tooth itself is *now* visibly longer than the surrounding ones, and loose; pressure makes the patient feel as if it were about to start from its socket. All these are evidences of suppuration at the root of the tooth, and if it be now extracted, a drop of matter will be seen attached to its root. In case of intense inflammation of the tooth-fang, the process of inflammation may not be terminated by the formation of matter; inflammation proceeds outwardly to the gum, the alveolus is absorbed, and a portion of the matter formed at the base of the tooth is thus evacuated, when the opening of the gum closes for a short time, until the matter again accumulates. Thus a sort of fistula is formed which can only be healed by the extraction of the tooth. The mischief may not be confined to the root of the tooth alone—which becomes absorbed at its extreme point and roughened —but may also extend to the jaw-bone, and render it carious. It sometimes happens that the cyst containing the pus at the root of the tooth, becomes changed into a mass of pappy consistence, which, comes away with the tooth on the latter being extracted. The treatment of this variety of odontalgia must be strictly antiphlogistic. Should the local application of cold fail in completely removing all the symptoms, leeches must be at once and freely applied to the gums. Where suppuration seems inevitable, a gently diaphoretic treatment with fomentations of warm water, or warm decoction of poppies, as marshmallow, or a solution of extract of henbane, in the proportion of five or ten grains to four ounces of warm water, will be found to assist materially the maturation of the abscess; as soon as the pus has been evacuated, the diseased tooth must be extracted.—It very often happens that a great number of the teeth are loosened, without any mechanical cause; this may depend either upon a sub-inflammatory affection of the lining membrane of the alveolar process, or upon that form of cynanche, denominated "Parotidea." In the latter instance time alone will effect a cure; the former requires for its cure repeated application of leeches over the affected portions of the alveolar process.—*Ibid* from *Wiener's Zeitschrift.*

